# *Salmonella enterica* serovar Choleraesuis vector delivering SaoA antigen confers protection against *Streptococcus suis* serotypes 2 and 7 in mice and pigs

**DOI:** 10.1186/s13567-017-0494-6

**Published:** 2017-12-21

**Authors:** Yu-an Li, Zhenying Ji, Xiaobo Wang, Shifeng Wang, Huoying Shi

**Affiliations:** 1 College of Veterinary Medicine, Yangzhou University, Yangzhou, 225009 Jiangsu China; 2 Jiangsu Co-innovation Center for the Prevention and Control of Important Animal Infectious Diseases and Zoonoses, Yangzhou, 225009 China; 3 Department of Infectious Diseases and Pathology, College of Veterinary Medicine, University of Florida, Gainesville, FL 32611-0880 USA

## Abstract

*Streptococcus suis* is one of the major pathogens that cause economic losses in the swine industry worldwide. However, current bacterins only provide limited prophylactic protection in the field. An ideal vaccine against *S. suis* should protect pigs against the clinical diseases caused by multiple serotypes, or at least protect against the dominant serotype in a given geographic region. A new recombinant *Salmonella enterica* serotype Choleraesuis vaccine vector, rSC0011, that is based on the regulated delayed attenuation system and regulated delayed antigen synthesis system, was developed recently. In this study, an improved recombinant attenuated *Salmonella* Choleraesuis vector, rSC0016, was developed by incorporating a *sopB* mutation to ensure adequate safety and maximal immunogenicity. In the spleens of mice, rSC0016 colonized less than rSC0011. rSC0016 and rSC0011 colonized similarly in Peyer’s patches of mice. The recombinant vaccine rSC0016(pS-SaoA) induced stronger cellular, humoral, and mucosal immune responses in mice and swine against SaoA, a conserved surface protein that is present in many *S. suis* serotypes, than did rSC0011(pS-SaoA) without *sopB* or rSC0018(pS-SaoA), which is an avirulent, chemically attenuated vaccine strain. rSC0016(pS-SaoA) provided 100% protection against *S. suis* serotype 2 in mice and pigs, and full cross-protection against SS7 in pigs. This new vaccine vector provides a foundation for the development of a universal vaccine against multiple serotypes of *S. suis* in pigs.

## Introduction

*Streptococcus suis* (*S. suis*) strains are classified into 35 serotypes based on the capsular polysaccharide antigens. Globally, the predominant *S. suis* serotype isolated from clinical cases of the disease in pigs is serotype 2, followed by serotypes 9, 3, 1/2, and 7, together with 15.5% nontypable strains [1]. To date, only vaccination with *S. suis* bacterins is performed to prevent *S. suis* infection in swine. Although several studies have demonstrated homologous protection [2], the procedure usually has a high rate of failure because the antigenicity of the vaccine is damaged by heat and formalin processing, which leads to the production of antibodies that are not associated with protection and/or are cross-reactive [3, 4]. Current vaccine research for *S*. *suis* focuses on subunit vaccine based on conserved proteins among different serotypes to protect pigs against clinical diseases caused by various *S. suis* serotypes, or protect against the dominant serotype in a given geographic region. However, the cost of many vaccines made from conserved proteins in veterinary medicine and swine medicine is relatively high compared to traditional bacterins.

To date, at least 24 subunit vaccine candidates have been identified [5–22]. Most of them were tested in mouse models [5, 8, 11, 12, 15, 17–22], and only a few were evaluated in pigs [5, 7, 9, 10, 12, 14, 16, 19, 23, 24]. The results showed that the protection conferred in swine is usually lower than that conferred in mice [10, 23]. Therefore, it is necessary to evaluate the candidate antigen in the target animal, pig. Moreover, the capacities to induce cross-protection were evaluated for a few antigens. Of these antigens, the surface-anchored protein (Sao) is a membrane-anchored bacterial protein reacting with 28 of 33 *S. suis* serotypes and 25 of 26 serotype 2 isolates, suggesting that it is a highly conserved protein in *S. suis* [16]. The effectiveness of Sao as a vaccine candidate depends upon the adjuvant used. Sao formulated with Emulsigen-Plus^®^ provided no protection against *S. suis* serotype 2 (SS2) because the induced antibodies lacked opsonizing activity against SS2 [16], whereas Sao formulated with the Quil-A adjuvant partially protected pigs against aerosol challenge with SS2 because it induced opsonizing antibodies [24]. So far, Sao is the only protein that has been shown to induce cross-protection against serotype 1 and 7 strains in mice and pigs [19]. These results indicate that the efficacy of the protection induced by Sao as a subunit vaccine candidate may depend on the choice of appropriate vector or adjuvant.

*Streptococcus suis* is an encapsulated microorganism. Host protection against infection by *S*. *suis* is primarily mediated by opsonophagocytosis, which is mainly associated with a Th1-type immune response [25]. *Salmonella* has excellent adjuvant attributes and induces high mucosal, cellular and humoral responses [26]. However, live attenuated *Salmonella*, traditionally generated by deletion of its virulence genes, can lack a balance between the loss of its disease-causing ability and the ability to persist and induce immune responses [27], and is therefore not an ideal vaccine vector. In addition, constitutive high-level antigen synthesis causes a metabolic burden to the vaccine vector strain that can reduce the vaccine strain’s ability to interact with host lymphoid tissues, resulting in a compromised immune response [28]. To solve these problems, the regulated delayed attenuation system was developed to make a strain displaying features of wild-type virulent strains of *Salmonella* at the time of immunization to enable strains to effectively colonize lymphoid tissues and then exhibit a regulated delayed attenuation in vivo to preclude inducing disease symptoms [29]. The regulated delayed antigen synthesis system could regulate antigen gene expression and permit high levels of antigen synthesis only after the vaccine strain reaches its target tissues [30]. The regulated delayed attenuation strategy includes a smooth-to-rough phenotypic change in lipopolysaccharides (LPS) with the presence or absence of mannose [31], and the replacement of the promoters of some virulence genes (*fur*, *crp*, and *phoPQ*) with a tightly regulated *araC* P_BAD_ cassette so that the expression of these genes is dependent on arabinose supplement during in vitro growth [29]. Following the colonization of the lymphoid tissues, these virulence-associated proteins cease to be synthesized because no mannose or arabinose is present in vivo [32]. Therefore, attenuation manifests gradually in vivo, precluding the induction of disease symptoms and inducing the desired antigen-specific immune responses [33]. Furthermore, an *araC* P_BAD_ cassette was used to regulate the expression of chromosomal *lacI* repressor gene, which binds to P_trc_ on an expression plasmid and blocks antigen synthesis in the presence of arabinose in vitro. Once *Salmonella* reaches arabinose-free, host immunocompetent sites, the concentration of LacI decreases with each cell division, allowing upregulation of antigen synthesis and induction of desired antigen-specific immune responses [30].

In a previous study, a recombinant attenuated *Salmonella enterica* serotype Choleraesuis (*Salmonella* Choleraesuis) vaccine strain rSC0011 with the regulated delayed strategy in the wild-type *Salmonella* Choleraesuis C78-3 background was constructed [34]. Strain rSC0011, carrying 6-phosphogluconate dehydrogenase (6-PGD) gene of SS2, conferred 100% protection against an SS2 challenge in mice [34], but less than 50% protection in pigs (unpublished data). To increase the efficacy of our vaccine strain, a *sopB* mutation was introduced into rSC0011 vector to generate a new recombinant attenuated *Salmonella* Choleraesuis strain rSC0016 based on the previous report that the *sopB* mutation could improve the immunogenicity of *Salmonella* vaccine vector in mice [35]. The heterologous antigen gene, *saoA* from *S*. *suis*, was cloned into the Asd^+^ expression plasmid pYA3493, generating plasmid pS-SaoA, which was used to transform the vector strain rSC0016, generating the candidate vaccine rSC0016 (pS-SaoA). The virulence and immune attributes of rSC0016 (pS-SaoA) in mice and piglets, and its cross-protection against SS2 and SS7 in piglets were evaluated. Our results showed that the improved recombinant attenuated *Salmonella* Choleraesuis, which combines the regulated delayed strategy and the *sopB* deletion, is more immunogenic than the parent strain rSC0011. It induces cross-protection against multiple serotypes of *S. suis* when delivering the heterologous protective antigen Sao. This could be a candidate for a universal vaccine against *S*. *suis*.

## Materials and methods

### Ethics statement

All animal experiments were approved by the Jiangsu Administrative Committee for Laboratory Animals (permission number SYXK-SU-2007-0005) and complied with the Jiangsu Laboratory Animal Welfare and Ethics guidelines of the Jiangsu Administrative Committee of Laboratory Animals.

### Strains, plasmids and culture conditions

The strains and plasmids used in this study are described in Table 1. *E. coli* strain χ7213, plasmid pRE112, and pYA3493 were provided by Dr Roy Curtiss 3^rd^ *S*. *suis* serotype 2 (SS2, CVCC3928) and *S*. *suis* serotype 7 (SS7) were purchased from China Institute of Veterinary Drug Control (Beijing, China). Strain C500, a government-approved live attenuated *Salmonella* Choleraesuis vaccine strain, was used as an attenuation control [36, 37]. Plasmid pYA3493 is an Asd^+^ vector, and plasmid pS-SaoA, derived from pYA3493, carries a *saoA* gene from SS2. LB medium [38], MacConkey agar (Difco), Nutrient broth (NB) and agar (Difco), and minimal salts medium and agar [39] were used for routine phenotype characterization. Nutrient broth (NB) and agar (Difco) are devoid of arabinose and mannose. When required, media were supplemented with chloramphenicol (Cm; 25 µg/mL), kanamycin (Kan; 50 µg/mL), 2,6-diaminopimelic acid (DAP; 50 µg/mL), l-arabinose (0.2% wt/vol), d-mannose (0.2% wt/vol), d-lactose (1% wt/vol), d-maltose (1% wt/vol), or sucrose (5% wt/vol). Selenite broth or Tetrathionate broth (Difco), with or without supplements, was used for enrichment of *Salmonella* Choleraesuis from animal tissues. Strains were grown and prepared as previously described [34]. Briefly, *Salmonella* Choleraesuis vaccine vector strains harboring plasmid pS-SaoA (expression vector) or pYA3493 (empty vector) were grown in LB broth with 0.2% arabinose and 0.2% mannose. SS2 or SS7 were cultured on brain heart infusion (BHI) agar containing 5% sheep blood or in Todd-Hewitt broth plus 0.5% yeast extract (Oxoid, Nepean, Ontario, Canada) [40].

**Table 1:** **Strains and plasmids**

Strain or plasmid	Relevant characteristics or genotype	Source or references
*E. coli* strains		
BL21	F^−^ *pT hsdSB*(rB^−^ mB^−^ *al dcm* (DE3)	Invitrogen
χ7213	*thi*-*1 thr*-*1 leuB6 fhuA21 lacY1 glnV44 asdA4 recA1 RP4 2*-*Tc::Mu pir; Km* ^*r*^	Dr Curtiss gift
*Salmonella* Choleraesuis		
C78-3	Wild type, virulent, CVCC79103	[65]
C500	*Salmonella* Choleraesuis vaccine strain attenuated by chemical mutation, CVCC79500	[37]
rSC0010	ΔP_crp527_::TT *araC* P_BAD_ *crp* Δ*pmi*-*2426* Δ*relA199::araC* P_BAD_ *lacI* TT	[34]
rSC0011	ΔP_crp527_::TT *araC* P_BAD_ *crp* Δ*pmi*-*2426* Δ*relA199::araC* P_BAD_ *lacI* TT Δ*asdA33*	[34]
rSC0013	rSC0010 Δ*sopB1686*	This work
rSC0016	rSC0013 Δ*asdA33*	This work
rSC0017	C78-3 Δ*sopB1686*	This work
rSC0018	C500 Δ*asdA33*	This work
*Streptococcus suis* serotype 2	Wild-type, virulent, CVCC3928	Lab stock
*Streptococcus suis* serotype 7	Wild-type isolated in field	Lab stock
Plasmids		
pYA3493	Plasmid Asd + ; pBR *ori*, β-lactamase signal sequence-based periplasmic secretion plasmid	[46]
pET28a	expression vector, T7 promoter; Km^r^	Novagen
pRE112	*oriT oriV sacB* Cm^R^	[34]
pS006	suicide vector for Δ*sopB1686*	This work
pYA3736	suicide vector for Δ*asdA33*	pRE112
pET28a-saoA	expression vector for purification of Hig-tag SaoA, T7 promoter, Km^+^	pET28a
pS-SaoA	pYA3493 with SaoA	pYA3493

### Construction and characterization of *Salmonella* Choleraesuis mutant strains

**Figure 1:**
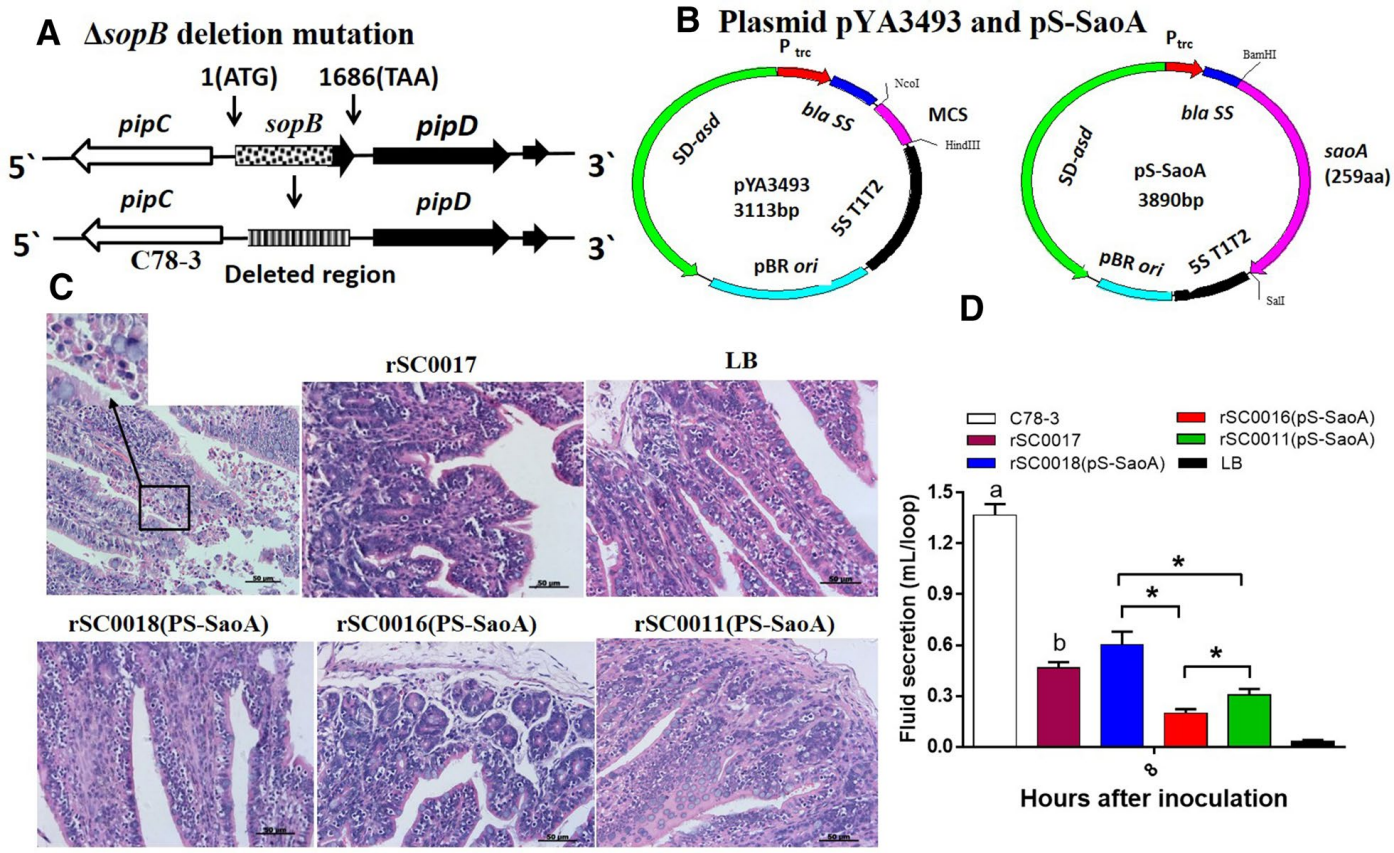
**Diagrams of chromosomal mutation, plasmid maps and phenotypic feature of the** ***sopB*** **mutant in** ***Salmonella*** **Choleraesuis strains. A** Chromosomal map of Δ*sopB* deletion mutation. **B** Empty plasmid vector pYA3493 and expression vector pS-SaoA. **C** Histopathological observations of rabbit ileal loops injected with different *Salmonella* Choleraesuis strains or LB control for 8 h. Bar: 50 μm. The small square showed the neutrophils in the submucosa of rabbit ileal injected with wild-type C78-3 strain. **D** Fluid secretion induced by *Salmonella* Choleraesuis strains in rabbit ileal loops. a, *P* < 0.05, wildtype *Salmonella* Choleraesuis C78-3 compared with LB, rSC0017, rSC0018(pS-SaoA), rSC0016(pS-SaoA) and rSC0011(pS-SaoA); b, *P* < 0.05, *Salmonella* Choleraesuis with Δ*sopB* mutation rS0017 compared to LB, rSC0018(pS-SaoA), rSC0011(pS-SaoA) and rSC0016(pS-SaoA); *P < 0.05, for the indicated strains were compared each other.

Five mutations were introduced into wild-type *Salmonella* Choleraesuis C78-3 by conjugation with *E. coli* χ7213 harboring different suicide vectors as previously described [29, 33, 34]. Plasmid constructs and primers for Δ*pmi*, ΔP_crp_::TT *araC* P_BAD_ *crp*, Δ*relA*::*araC* P_BAD_ *lacI* TT, Δ*sopB,* and Δ*asdA* have been described [34, 41]. The upstream primers of *sopB* mutation in *Salmonella* Choleraesuis (P1: 5′-CGCAGAGCTCATATCACCTATAATTATC-3′; P2: 5′- ATTAGGTACCAGCAGTATTGTCTGCGTCAGC -3′) (Figure 1A) have not been previously described; the upstream sequences are different between *Salmonella* Choleraesuis and *Salmonella* Typhimurium.

To detect the effect of *sopB* deletion, the fluid secretion in rabbit ileal loops after inoculation with wild-type strain C78-3, rSC0017 (*Salmonella* Choleraesuis C78-3 with *sopB* mutation), vaccine strains rSC0018(pS-SaoA), rSC0011(pS-SaoA), and rSC0016(pS-SaoA) were compared as previously described [41]. Briefly, 38-week-old New Zealand White rabbits were fasted overnight and anesthetized with isoflurane by an endotracheal tube. The ileum was exposed and ligated into several loops of 5–6 cm long with 1 cm spacers. 1 mL of indicated strains, containing around 1 × 10^9^ colony-forming units (CFU), were injected into separate loops. LB medium was injected into one of the loops as a control. The abdominal musculature was closed using 3–0 chromic gut sutures and the skin closed with 3–0 ethilon sutures. Rabbits were maintained in a thermal blanket at 37 °C. After 8 h, the rabbits were euthanized with an overdose of sodium pentobarbital. The abdomen was reopened and the fluid within the ligated loops was collected and measured. The loops were fixed in 10% formalin and subjected to histopathological examination.

All mutations were verified by PCR using corresponding primers. The ΔP_crp527_::TT *araC* P_BAD_ *crp* deletion-insertion mutation was confirmed by growing on MacConkey maltose agar with or without 0.2% arabinose [29]. LPS profiles were examined by silver staining in 12% sodium dodecyl sulfate–polyacrylamide gel for Δ*pmi* mutation [34]. LacI production for Δ*relA*::*araC* P_BAD_ *lacI* TT mutation was confirmed by Western blot [34]. The Δ*asdA* mutation was confirmed by growth with or without DAP in LB media [34].

### Construction and analysis of the plasmid stability of the attenuated *Salmonella* Choleraesuis vector with plasmids pYA3493 and pS-SaoA

Primers for *S*. *suis saoA* gene were designed according to GenBank accession no. GI:8151617 (*saoA*-F:ATGGATCCCAACCTGATGGGGGAC; *saoA*-R: GCGTCGACCATTGCTTCCTTAGAG) to amplify N-terminal (121–1593 bp) of *saoA* gene [42]. The DNA of *saoA* either from SS2 or SS7 was amplified by PCR, purified by AxyPrep TM PCR Cleanup kit (Axygen, Union, CA, USA), and cloned into the expression vector pET28a to generate plasmid pET28a-SaoA according to the manufacturer’s instructions (Novagen, Darmstadt, Germany). The identity of the insert in pET28a was verified by DNA sequence analysis. Following digestion of plasmids pET28a-SaoA and pYA3493 with *Bam*HI and *Sal*I, the *saoA* from SS2 was cloned into pYA3493 to generate pS-SaoA. pS-SaoA and empty vector pYA3493 were transformed into the live attenuated *Salmonella* Choleraesuis (Figure 1B).

To evaluate the production of SaoA in live attenuated *Salmonella* Choleraesuis, sodium dodecyl sulfate polyacrylamide gel electrophoresis (SDS-PAGE) was performed as described [33]. Strains harboring plasmid pS-SaoA or control vector pYA3493 were grown in LB medium with indicating supplements (0.2% arabinose and mannose) at 37 °C with aeration. When the culture reached an OD_600_ of 0.8, Isopropyl-d-thiogalactopyranoside (IPTG) was added to the culture, and the culture continued to grow for 3 h. The bacteria numbers were normalized by OD_600_. 1 mL of culture was collected for Western blot analysis using anti-SaoA antiserum as previously described [33]. Briefly, total protein samples were separated on a 10% (wt/vol) SDS-PAGE gel and transferred onto nitrocellulose membranes. Phosphate buffered saline (PBS) with 5% fat-free milk powder and 0.05% Tween 20 (PBS-T) was used for blocking. The membrane was incubated with an appropriate anti-rabbit polyclonal antibody (1:10 000, anti-SaoA) or anti-GroEL (Sigma, St. Louis, MO, USA) for 1 h at room temperature, washed three times with PBS-T, and then horseradish peroxidase-conjugated goat anti-rabbit immunoglobulin G was used as the secondary antibody (Sigma). The membrane was developed with a chemiluminescent substrate using the kit ECL Plus Western Blotting System (GE Healthcare, Chalfont St Giles, UK) according to manufacturer’s instructions. Densitometry was quantified using Image J software (Image J2) [43].

To examine the stability of plasmids pS-SaoA and pYA3493 in *Salmonella* Choleraesuis vector, the strains containing pS-SaoA or pYA3493 were cultured with the daily passage of 1:1000 dilutions for 5 consecutive days (about 50 generations) in LB medium with DAP, arabinose, and mannose. At the 50th generation, Asd^+^ plasmids pS-SaoA and pYA3493 were tested by endonuclease digestion. The mutations of the live attenuated *Salmonella* Choleraesuis vector were confirmed by PCR. SaoA production was evaluated by Western blot.

### Preparation the SaoA protein of SS2 or SS7, antiserum to the SaoA protein of SS2, and *Salmonella* outer membrane proteins (SOMPs) of *Salmonella* Choleraesuis

*Escherichia coli* strain BL21 carrying pET28a-SaoA was used for the synthesis of the His-tagged SaoA fusion protein (Table 1). Cells were grown to mid-log phase (OD_600_ of 0.6) in LB medium with kanamycin at 37 °C and induced with 1 mM IPTG for 3 h. The His-tagged SaoA protein was purified by using a CelLytic B Plus kit (Sigma) and a His-Select nickel affinity gel (Sigma) according to manufacturer’s instructions. To create rabbit antibodies to SaoA, two female New Zealand White rabbits (8 weeks old) were injected subcutaneously with an emulsion consisting of a 1:1 ratio of 400 μg of the His-tagged SaoA protein to complete Freund adjuvant (Sigma). Two weeks after the primary injection, the immunization was repeated but with incomplete Freund adjuvant (Sigma) instead of complete adjuvant. Three weeks after the second immunization, the rabbits were boosted with 200 μg of the His-tagged SaoA protein without adjuvant. Two weeks after the third immunization, the rabbits were bled to obtain anti-SaoA rabbit antiserum [34].

*Salmonella* outer membrane proteins (SOMPs) were prepared from wild-type *Salmonella* Choleraesuis C78-3. Briefly, total envelope pellets were suspended in 4 mL of 20 mM Tris–HCl (pH 8.6) containing 1% Sarkosyl and incubated for 30 min on ice. The outer membrane fraction was obtained as a pellet after centrifugation at 132 000 × *g* at 4 °C for 1 h. The pellet was re-suspended in 4 mL of 20 mM Tris–HCl buffer (pH 8.6) and stored at −20 °C.

### Distribution of *Salmonella* bacteria in BALB/c mice

Colonization assay for mutant strain was carried out as described [34]. 140 7-week-old female BALB/c mice were divided into seven groups with 20 mice per group. Groups of mice were orally inoculated with 20 μL buffered saline with gelatin (BSG) containing 1 × 10^9^ CFU of *Salmonella* strains. Liver, spleen, and Peyer’s patches of mice were collected on days 3, 7, 14, and 21 post-infection. Tissues were weighed and homogenized in a final volume of 1 mL BSG [39]. Serial dilutions were plated onto MacConkey agar plates containing 1% lactose, with or without 0.2% arabinose and 0.2% mannose, to determine the number of viable bacteria. Plates were incubated at 37 °C for at least 18 h. The residual 900 mL of homogenized tissues were inoculated into 5 mL Tetrathionate Broth (Difco) for *Salmonella* enrichment when no colonies were observed on the plates. Samples that were negative by direct plating and positive by enrichment were recorded as 10 CFU/g. Samples that were negative by both direct plating and enrichment were recorded as 0 CFU/g [39]. The assay was performed twice, and the data were pooled.

### Immunization of mice and piglets

#### Immunization of mice

*Salmonella* Choleraesuis vector strains harboring plasmids pS-SaoA or pYA3493 (empty vector) were grown in LB broth with 0.2% arabinose and 0.2% mannose overnight at 37 °C as standing cultures that were diluted 1:100 in the same medium. The culture was grown with aeration (180 rpm) at 37 °C to an OD_600_ of 0.85–0.9. Bacteria were collected by centrifugation at room temperature and re-suspended in BSG. 15 7-week-old BALB/c female mice were inoculated orally with 20 μL BSG containing 1 ± 0.3 × 10^9^ CFU of *Salmonella* Choleraesuis vectors with pS-SaoA or plasmid pYA3493 or 20 μL BSG control. Mice were boosted with the same dose of the same strain 3 weeks later. About 100 μL of whole blood was obtained by mandibular vein puncture 3 weeks after primary inoculation and 2 weeks after boosting. Serum was removed from the whole-blood samples and stored at −20 °C. Vaginal-wash samples in mice were collected at the indicated time and stored at −20 °C as described [33, 34]. Ten mice from each group were challenged with SS2, and five mice were used for cytokine detection. This experiment was performed twice, with each group (15 mice) receiving approximately the same dose of vaccine. The results from both experiments were similar and the data were pooled for analysis.

#### Immunization of piglets

The preparation of the inoculum of *Salmonella* Choleraesuis vector strains harboring plasmids pS-SaoA or pYA3493 was performed as described in a previous section. Seventy-five 3-week-old castrated piglets were purchased from a commercial pig farm in Jiangsu Province, China. To select the pigs with negative antibody titers against SS2, SS7, and *Salmonella* Choleraesuis, blood was obtained from pigs by cranial vena cava and incubated at 37 °C for 60 min. The resulting clot was pelleted by centrifugation. Serum was removed from the whole-blood sample and stored at −20 °C. Serum IgG responses to SS2 SaoA, SS7 SaoA, and *Salmonella* Choleraesuis outer membrane proteins (OMPs) were measured by enzyme-linked immunosorbent assay (ELISA) (see below) [33, 44, 45]. The IgG titer that was less than cut-off value was considered as seronegative. These piglets were randomly assigned to five groups with 15 piglets/group, named rSC0016(pS-SaoA) group, rSC0016(pYA3493) group, rSC0018(pS-SaoA) group, rSC0018(pYA3493) group, and BSG group. Piglets were inoculated orally with 10 mL 1 ± 0.3 × 10^9^ CFU of *Salmonella* Choleraesuis vector strains carrying either the SaoA expression plasmid pS-SaoA or empty plasmid pYA3493 or BSG control. Piglets were boosted with the same dose of the same strain 3 weeks later. Serum and nasal swabs were collected at 3, and 5 weeks after first immunization [10, 16]. In each group, five piglets were challenged with SS2 and five with SS7 at 2 weeks after a boost. Five piglets were used to detect cytokine levels in the blood and spleen at different time points (0.5, 3, 5, and 7 days after boost). This experiment was performed twice with each group receiving approximately the same dose of vaccine. The results from both experiments were similar and the data were pooled for analysis.

### ELISA test

Serum IgG responses to SaoA, to OMPs of *Salmonella* Choleraesuis in mice and pigs, and vaginal wash IgA antibody to SaoA in mice and nasal cavity wash IgA in pigs against SaoA were measured by ELISA. The initial dilutions of serum or vaginal/nasal wash from an individual animal were 1:50 or 1:10, respectively. Briefly, 100 µL solutions containing either 1 μg/well of *Salmonella* OMPs or of SaoA in sodium carbonate-bicarbonate coating buffer (pH 9.6) was used to coat Nunc-Immuno MaxiSop 96-well plates (Corning, NY, USA). The plates were then incubated overnight at 4 °C. The next day, the plates were washed 3 times with PBST (PBS with 0.1% Tween 20) and then blocked with a PBST containing 2% BSA solution for 2 h at room temperature. A 100 μL volume of serially diluted sample was added in triplicate to individual wells, and the plates were incubated for 1 h at room temperature. After washing wells with PBST, goat anti-mouse or goat anti-pig IgG-horseradish peroxidase (HRP) detection antibody (Sigma, St. Louis, MO, USA) diluted 1:5000 in PBST was added to the wells and the plate was incubated at room temperature for 60 min with gentle rocking. Plates were developed with 1-Step TM Ultra TMB-ELISA (Thermo Fisher Scientific, Waltham, MA, USA) and quenched with 3N H_2_SO4. Absorbance was recorded at 450 nm using an automated ELISA plate reader (model EL311SX; Biotek, Winooski, VT). Absorbance readings 2.1 times higher than the baseline values of preimmune sera (negative control: about 0.045–0.052) were considered positive [33, 44–46].

Sera were collected from mice or piglets on 0.5, 3, and 5 days. In addition, spleen tissues were removed at 7 days after boost immunization. Spleens were homogenized in a final volume of 1 mL (mouse tissues) or 5 mL (pig tissues) BSG at a ratio of weight: volume of 1:9. The supernatants were stored at −70 °C. Cytokines in sera and spleen of mice or pigs were analyzed by sandwich ELISA using commercial kits (Abcam, Cambridge, UK, except pig-specific IL4 ELISA kit, which was purchased from Thermo Fisher Scientific) according to manufacturer’s protocols.

### Analysis of the cross-reactivity between SS2 and SS7 in pigs

Since the *saoA* gene was from SS2, the cross-reactivity of sera from pigs immunized with rSC0018(pS-SaoA) or rSC0016(pS-SaoA) was checked against total cell lysates from SS2 or SS7, respectively. Total *S. suis* proteins (SS2 or SS7 strain) were extracted from bacterial culture and 10 μg of proteins were separated by 12% SDS-PAGE gel electrophoresis and transferred to nitrocellulose membrane. The membrane was incubated with immunized pig serum (1:100 dilution) for 1 h at room temperature and subsequently washed three times with 0.05% w/v Tween-20 in TBS for 5 min. Anti-pig IgG conjugated to horseradish peroxidase (1:5000 dilution, Sigma) was used as secondary antibodies. The membrane was developed with a chemiluminescent substrate using the kit ECL Plus Western Blotting System (GE Healthcare) according to manufacturer’s instructions.

### Challenge experiment with SS2 in mice or SS2 and SS7 in pigs

Ten mice were challenged with 3 × 10^8^ CFU of SS2 intraperitoneally (i.p.) at 2 weeks after the booster. The 50% lethal dose (LD_50_) of SS2 in BALB/c mice was 1.2 × 10^7^ CFU. Challenged mice were monitored daily for 15 days [33].

Five pigs in each group were challenged with SS2 by intravenous injection (i.v.) with 5.0 × 10^8^ CFU (LD_50_ of SS2 in pigs was 5.6 × 10^7^ CFU). The clinical symptoms and survival of pigs were monitored for 14 days. When needed, the brain of pigs was fixed in 10% formalin buffer (pH 7.2). Thin sections (5 mm) of tissue were stained with hematoxylin/eosin and examined by light microscopy to detect histological changes caused by SS2. Another five pigs in each group were challenged by i.v. with 1.0 × 10^10^ CFU of SS7. The rectal temperatures of pigs challenged with SS7 were measured at 0, 1, 2, 3, 4, 5, 6, and 7 days post-inoculation [47].

### Statistical analysis

Data were presented as the geometric means and standard deviations for all assays. The Mann–Whitney U test (GraphPad Software, Inc., San Diego, CA) was used to evaluate the persistence of strains in vivo and antibody levels of mice. The Kaplan–Meier method (SPSS software) was applied to obtain the survival fractions following i.p. challenges of immunized mice and i.v. challenges of immunized pigs. A *P* value of 0.05 was considered statistically significant.

## Results

### Construction of *sopB* deletion in *Salmonella* Choleraesuis vaccine vectors

Previous reports have shown that the inactivation of *sopB* in *Salmonella* Typhimurium improves humoral and cellular immunity in the host [35, 48]. To improve the safety and increase the immunogenicity of *Salmonella* Choleraesuis vector, a *sopB* mutation was introduced into wild-type *Salmonella* Choleraesuis C78-3 to generate strain rSC0017 (Figure 1A) or into *Salmonella* Choleraesuis rSC0013 to generate strain rSC0016 (Table 1). Strain rSC0016 has the mutations for the regulated delayed attenuation system and the regulated delayed antigen synthesis system, characterized by ΔP_crp_::TT *araC* P_BAD_ *crp*, Δ*pmi*, and Δ*relA*::*araC* P_BAD_ *lacI* TT [34], in addition to the *sopB* mutation. It also has a Δ*asdA* mutation for the balanced-lethal system to facilitate its use as a vector.

### Phenotypic characterization of live attenuated *Salmonella* Choleraesuis vaccine vector with *sopB* mutation

SopB plays a role in water efflux, fluid secretion, and subsequent diarrhea associated with *Salmonella* infection [49]. In this study, the vaccine strains rSC0018(pS-SaoA), rSC0011(pS-SaoA), rSC0016(pS-SaoA), the strain rSC0017 with a *sopB* deletion mutation, the parental wild-type strain C78-3, and a LB control were injected into six parts of the ileal loop in rabbits to detect the effect of the *sopB* deletion in *Salmonella* Choleraesuis. Hematoxylin–eosin staining of ileal loop sections revealed populations of neutrophilic cells in the ileum loops infected by the wild-type strain C78-3, but not in those infected by rSC0017, rSC0018(pS-SaoA), rSC0011(pS-SaoA), or rSC0016(pS-SaoA). These loops were similar to the loops injected by LB (Figure 1C). The fluid accumulation in the ileal loops injected with the wild-type parental strain C78-3 was significantly higher than those injected with rSC0017, rSC0018(pS-SaoA), rSC0011(pS-SaoA), rSC0016(pS-SaoA) and LB (Figure 1D). Particularly, the inflammatory exudate in the ileal loops injected with rSC0016(pS-SaoA) containing *sopB* mutation was significantly reduced compared to that injected with rSC0011(pS-SaoA) without the *sopB* mutation. In addition, the fluid accumulation in the ileal loops injected with rSC0018(pS-SaoA) was significantly more than that in those injected with rSC0011(pS-SaoA) and rSC0016(pS-SaoA), even higher than that of rSC0017 (Figure 1D). Little fluid was detected in the loops injected with LB control, which was significantly less than those injected with C78-3, rSC0017, SC0018(pS-SaoA), rSC0011(pS-SaoA), and rSC0016(pS-SaoA) (Figure 1D).

**Figure 2:**
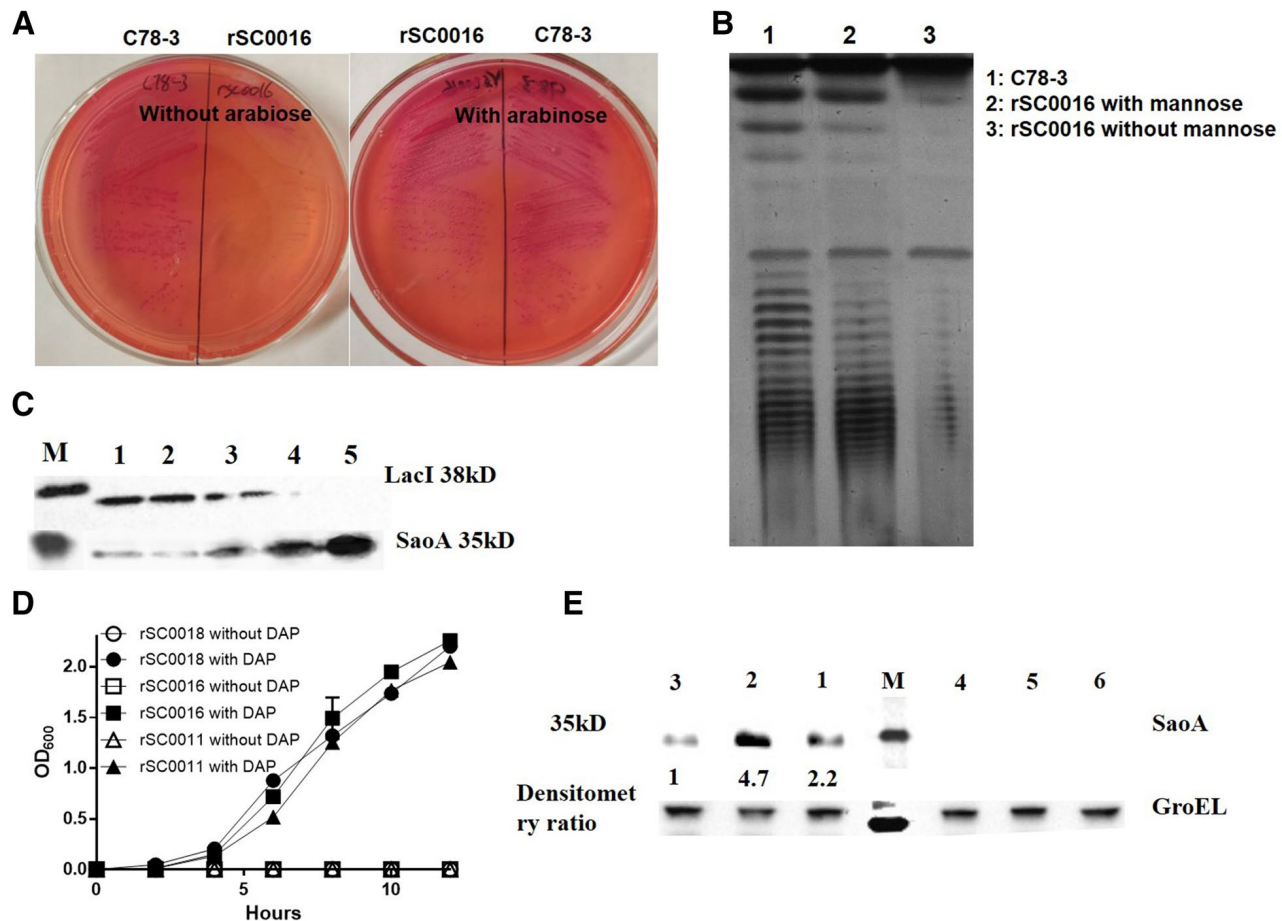
**Phenotypic characterization of** ***Salmonella*** **Choleraesuis strains rSC0016. A** Phenotype of ΔP_crp527_::TT *araC* P_BAD_ *crp*. Strain rSC0016 with the ΔP_crp527_::TT *araC* P_BAD_ *crp* mutation were grown on MacConkey maltose agar with and without 0.2% arabinose. **B** Phenotype of Δ*pmi* mutation. LPS profiles of Δ*pmi* mutant strains rSC0016 in NB grown with or without 0.2% mannose. Lanes: 1, wild-type C78-3; 2, rSC0016 with mannose; 3, rSC0016 without mannose. **C** Regulated decreased synthesis of LacI and regulated delayed synthesis of SaoA proteins in rSC0016(pS-SaoA) containing Δ*relA*::*araC* P_BAD_ *lacI* TT mutation. Strain rSC0016(pS-SaoA) were grown in NB with arabinose and mannose (Lane 1) and then diluted 1:10 into fresh NB without arabinose and mannose until OD_600_ to 0.8. The process was repeated for four times (Lane 2–5); each lane was loaded around 2.5 × 10^7^ CFU bacteria. Synthesis of LacI and SaoA were detected by Western blot using correspondent antiserum. M: protein marker. **D** Phenotype of Δ*asdA* mutation. Growth curves of rSC0016 in LB with and without DAP. Growth was monitored by measuring OD_600_ at the indicated time intervals. **E** Synthesis of SaoA in *Salmonella* Choleraesuis vector rSC0011, rSC0016 and rSC0018. An equal amount of cells was subjected to SDS-PAGE analysis. Immunoblot was detected with SaoA—specific polyclonal antibody. Densitometry ratio was quantified using Image J software. M: protein Marker; 1: rSC0011(pS-SaoA); 2: rSC0016(pS-SaoA); 3: rSC0018(pS-SaoA); 4: rSC0011(pYA3493); 5: rSC0016(pYA3493); 6: rSC0018(pYA3493); GroEL was used as a control.

The phenotype of ΔP_crp527_::TT *araC* P_BAD_ *crp* mutation was tested on MacConkey maltose agar with and without arabinose. The strain with ΔP_crp527_::TT *araC* P_BAD_ *crp* mutation formed red colonies on MacConkey maltose agar in the presence of arabinose, and white colonies in the absence of arabinose (Figure 2A) [29, 46]. The smooth LPS pattern was observed in strain rSC0016 grown with mannose, and rough without mannose due to the presence of Δ*pmi* mutation (Figure 2B) [34]. To verify the regulated delayed synthesis of heterologous protein, strain rSC0016(pS-SaoA) was passaged four times in arabinose -and mannose—free medium after it was grown in nutrient broth medium with 0.2% arabinose and mannose, as described previously [34]. The expected phenotype, the gradual disappearance of the LacI protein and the increased production of the heterologous protein SaoA, was observed in the strain rSC0016(pS-SaoA) (Figure 2C) [29, 46]. The introduction of the Δ*asdA* mutation into *Salmonella* Choleraesuis strains rSC0010, rSC0013, and C500 (Table 1) [46, 50] did not affect the growth of the resulting strains rSC0011, rSC0016, and rSC0018 (Figure 2D). However, the production of SaoA protein in rSC0011 and rSC0016 was 2.2-fold and 4.7-fold higher than that in rSC0018, respectively, whereas the production of SaoA protein in strain rSC0016 was 2.5-fold higher than that in strain rSC0011(pS-SaoA) (Figure 2E).

### Plasmids pYA3493 and pS-SaoA are stable in attenuated *Salmonella* Choleraesuis rSC0016 strain

A single colony of rSC0018, rSC0016 and rSC0011 either with pS-SaoA or pYA3493 was grown in LB with 0.2% mannose, 0.2% arabinose, and 50 µg/mL DAP for 50 generations [34]. All the colonies that were examined using PCR or endonuclease digestion contained either plasmid pS-SaoA or plasmid pYA3493, indicating that pS-SaoA and pYA3493 are equally stable in the vaccine strains (data not shown). These colonies can produce the expected SaoA protein (data not shown).

### Distribution of *Salmonella* Choleraesuis rSC0016 vector in BALB/c mice

**Figure 3:**
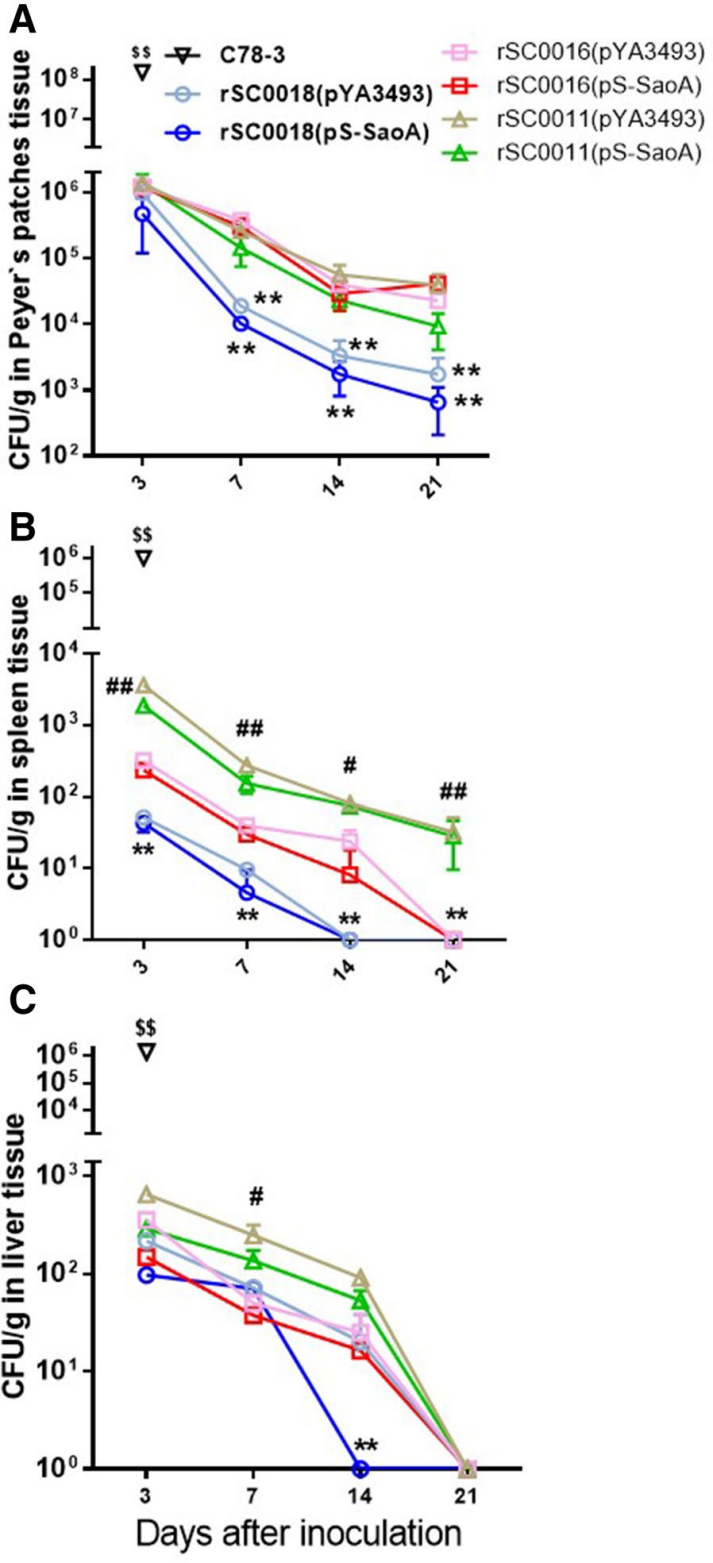
**Colonization of** ***Salmonella*** **Choleraesuis rSC0016 in BALB/c mice at different time points.** The numbers of *Salmonella* Choleraesuis C78-3, rSC0018(pYA3493), rSC0018(pS-SaoA), SC0016(pYA3493), rSC0016(pS-SaoA), rSC0011(pYA3493), and SC0011(pS-SaoA) in Peyer’s patches (**A**), spleen (**B**) and liver (**C**), of mice at 3, 7, 14, and 21 days after oral inoculation with 1.0 ± 0.3 × 10^9^ CFU of the indicated strains were plotted. Bars represent the arithmetic mean ± standard deviations from ten mice per group. **P < 0.01, for rSC0018 compared to rSC0016 or to rSC0011 with either pYA3493 or pS-SaoA, respectively; ^#^ *P* < 0.05, ^##^ *P* < 0.01, for rSC0011 compared to rSC0016 carrying either pYA3493 or pS-SaoA, respectively; ^$$^ *P* < 0.01, for C78-3 compared to rSC0018, rSC0016 and rSC0011 carrying either pYA3493 or pS-SaoA, as indicated. The data were collected from two independent experiments.

The *Salmonella* Typhimurium Δ*sopB* mutant is attenuated in its ability to cause gastroenteritis in calves [51], but not in its invasion of host tissues [52]. The distributions of wild-type *Salmonella* Choleraesuis C78-3, rSC0018(pYA3493), rSC0018(pS-SaoA), rSC0016(pYA3493), rSC0016(pS-SaoA), rSC0011(pYA3493), and rSC0018(pS-SaoA) in the Peyer’s patches, spleens, and livers of BALB/c mice were compared. The mice inoculated with wild-type *Salmonella* Choleraesuis C78-3 died 3 days after inoculation, whereas the mice infected orally with 10^9^ CFU of rSC0018(pYA3493), rSC0018(pS-SaoA), rSC0016(pYA3493), rSC0016(pS-SaoA), rSC0011(pYA3493), or rSC0011(pS-SaoA) survived without displaying any disease symptom. The bacteria titer of wild-type strain C78-3 in Peyer’s patches, spleen, and liver were significantly higher than those of strains rSC0016(pYA3493), rSC0016(pS-SaoA), rSC0011(pYA3493), rSC0011(pS-SaoA), rSC0018(pYA3493), and rSC0018(pS-SaoA) at 3 days after inoculation (Figures 3A–C). The three attenuated strains rSC0011, rSC0016, and rSC0018 with plasmid pS-SaoA or pYA3493 colonized Peyer’s patches equally well 3 days after infection, suggesting that the deletion of *sopB* did not affect the initial colonization of Peyer’s patches by *Salmonella* Choleraesuis vaccines. The titers of bacteria in Peyer’s patches were similar for strains rSC0016(pYA3493), rSC0016(pS-SaoA), rSC0011(pYA3493), and rSC0016(pS-SaoA) at 3–21 days post-inoculation. All of these strains colonized Peyer’s patches better than attenuated vaccine strains rSC0018(pYA3493) and rSC0018(pS-SaoA) did at 7, 14, and 21 days post-inoculation, with about 13-fold, 22.5-fold, and 60-fold higher titers, respectively (Figure 3A). In the spleen, the titer of bacteria of strains rSC0011(pYA3493) and rSC0011(pS-SaoA) was significantly higher than that of rSC0016(pYA3493), rSC0016(pS-SaoA), rSC0018(pYA3493), and rSC0018(pS-SaoA), respectively, at 3, 7, 14, and 21 days, respectively (Figure 3B). Furthermore, more rSC0016(pYA3493), rSC0016(pS-SaoA) colonized in spleen than that rSC0018(pYA3493) and rSC0018(pS-SaoA) did at 3, 7, and 14 days, respectively (Figure 3B). These results indicated that the *sopB* mutation reduced the colonization ability of *Salmonella* Choleraesuis vaccine strains in mice spleen. *Salmonella* vectors rSC0016 and rSC0011 with the regulated delayed strategy displayed better colonization in the spleen compared with rSC0018. In liver, the titers of rSC0016(pYA3493) and rSC0016(pS-SaoA) were similar to those of rSC0011(pYA3493), rSC0011(pS-SaoA), rSC0018(pYA3493), and rSC0018(pS-SaoA) at 3 days after inoculation (Figure 3C). At day 7 post-inoculation, the titers of rSC0016(pYA3493) and rSC0016(pS-SaoA) were 5.0-fold and fourfold lower than those of rSC0011(pYA3493) and rSC0011(pS-SaoA), respectively, whereas the strain rSC0018(pS-SaoA) was the fastest to be cleared in liver (Figure 3C). These results indicated that the *sopB* deletion slightly impaired the colonization in the liver of *Salmonella* Choleraesuis vaccine strains. Although the titers of rSC0011(pS-SaoA) and rSC0016(pS-SaoA) were slightly lower than those of rSC0011(pYA3493) and rSC0016(pYA3493) in Peyer’s patches, spleen, and liver, they were not significantly different (Figures 3A–C).

### *Salmonella* Choleraesuis vaccine vector with *sopB* mutation induces higher antibody responses in mice

**Figure 4:**
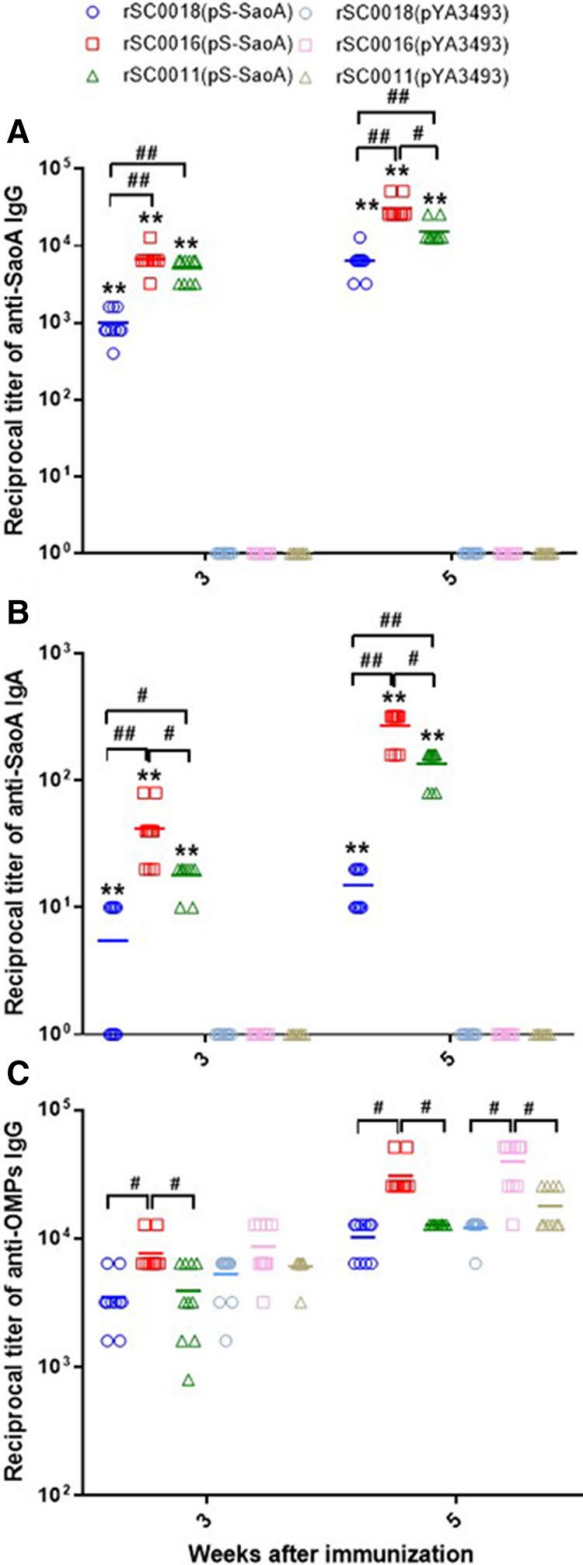
**Antibody responses in mice. A** Serum IgG responses to SaoA. **B** Vaginal IgA responses to SaoA and **C** Serum IgG to *Salmonella* Choleraesuis OMPs were measured by ELISA. The data represent reciprocal antibody titers in sera from ten mice orally immunized with attenuated *Salmonella* carrying either pS-SaoA or pYA3493 (empty vector) and BSG at indicated weeks after immunization. Serum and vaginal wash obtained from individual mice were serially diluted to obtain titers, starting from either 1:50 or 1:10. Error bars represent variation between mice. Significant differences were indicated. **P < 0.01, for *Salmonella* Choleraesuis with pS-SaoA compared to *Salmonella* Choleraesuis with pYA3493 in **A** and **C**; ^#^ *P* < 0.05; ^##^ *P* < 0.01, for *Salmonella* carrying either pS-SaoA or pYA3493 compared each other. No antibody responses were detected to antigen tested in mice immunized with the only BSG or in pre-immune sera from vaccinated mice. ELISA was performed twice with identical results.

To test the immunogenicity of *Salmonella* Choleraesuis vaccine strain that combines regulated delayed strategy and the *sopB* mutation, the antibody responses to SS2 SaoA and *Salmonella* Choleraesuis OMPs in mice were measured at 3 and 5 weeks after the primary immunization (Figures 4A, B and C). Higher serum IgG and vaginal IgA titers against SaoA were detected in the mice immunized with the live attenuated *Salmonella* Choleraesuis vaccines carrying plasmid pS-SaoA [rSC0016(pS-SaoA), rSC0011(pS-SaoA), and rSC0018(pS-SaoA)] compared to those with BSG or the strain containing the empty vector pYA3493 (Figures 4A and B). The levels of serum IgG against SaoA in mice immunized with rSC0016(pS-SaoA) were similar to those in mice immunized with rSC0011(pS-SaoA), yet significantly higher than those immunized with rSC0018(pS-SaoA) (Figure 4A). Higher vaginal IgA titers against SaoA were observed in the mice immunized with rSC0016(pS-SaoA) or rSC0011(pS-SaoA) compared to those with rSC0018(pS-SaoA) (Figure 4B). At 3 or 5 weeks after vaccination, the mucosal IgA responses against SaoA in mice immunized with rSC0016(pS-SaoA) were also significantly higher than those with rSC0011(pS-SaoA) (Figure 4B). At 3 weeks after the primary immunization, the IgG antibody responses to *Salmonella* Choleraesuis OMPs were significantly higher in mice immunized with rSC0016(pS-SaoA) than those with rSC0011(pS-SaoA) or rSC0018(pS-SaoA) (Figure 4C). At 5 weeks post-infection, higher IgG responses against *Salmonella* Choleraesuis OMPs were observed in mice immunized with rSC0016 carrying either pS-SaoA or pYA3493 than those with rSC0011 or rSC0018 (Figure 4C).

### *Salmonella* Choleraesuis vaccine vector with *sopB* mutation induces higher interferon γ (IFN-γ), interleukin 4 (IL-4), and interleukin 17A (IL-17A) responses in mice

**Figure 5:**
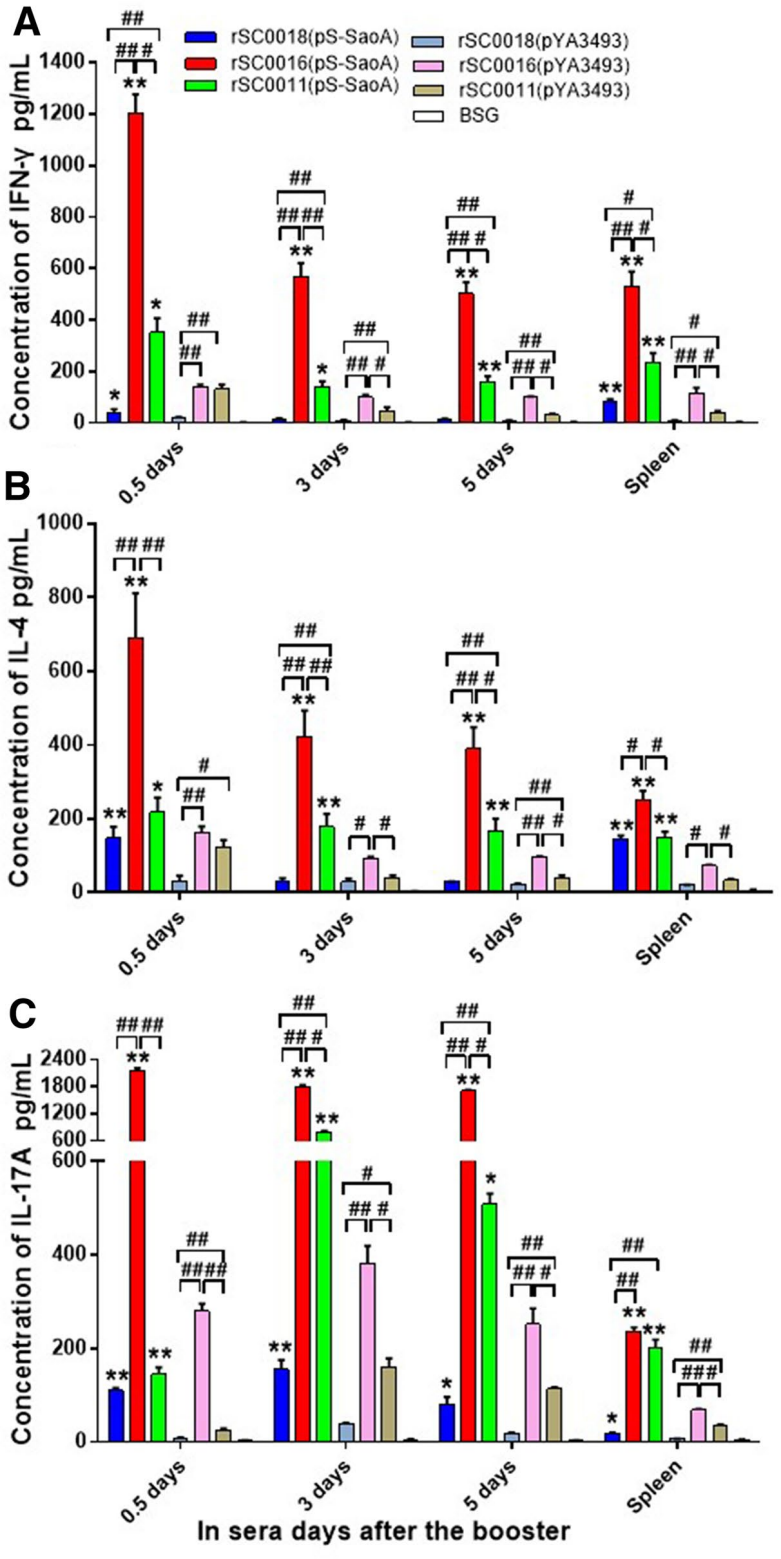
**Cytokines levels in ten mice immunized with** ***Salmonella*** **Choleraesuis vaccines.** IFN-γ (**A**), IL-4 (**B**) or IL-17A (**C**) in sera at 0.5, 3, and 5 days, in the spleen at 7 days after the boost were assayed with ELISA kit. BSG control was also included. *P < 0.05; **P < 0.01, for the cytokines levels induced by strains rSC0011, rSC0016 and rSC0018 containing pS-SaoA compared with that containing the empty vector pYA3493; ^#^ *P* < 0.05, ^##^ *P* < 0.01 for the significant differences between groups were indicated. The assay was performed in triplicate. The data were collected from two experiments and analyzed.

A previous study showed that the introduction of a *sopB* mutation into different attenuated *Salmonella* Typhimurium strains enhanced the immune responses to the delivered heterologous antigen in mice [48]. To further characterize the influence of *sopB* on immune responses, the levels of IFN-γ, IL-4, and IL-17A in mice sera collected at 0.5, 3, and 5 days and in the spleen collected at 7 days after the booster were measured. The concentrations of IFN-γ, IL-4, and IL-17A induced in mice immunized with vector rSC0016, rSC0011, or rSC0018 carrying either pYA3493 or pS-SaoA were significantly higher in the sera and spleen tissues than those induced in mice treated with BSG (Figures 5A–C, *P* < 0.01). The three vectors that contain pS-SaoA (rSC0011, rSC0016 and rSC0018) induced higher levels of IFN-γ, IL-4, and IL-17A than the strain with the empty vector pYA3493 in sera and spleen of mice (Figures 5A–C, *P < 0.05; **P < 0.01). Conversely, rSC0018(pS-SaoA) induced similar levels of IFN-γ and IL-4 in sera compared to those induced by rSC0018(pYA3493) at 3 and 5 days after boost immunization (Figures 5A and B).

Higher IFN-γ, IL-4, and IL-17A levels were produced in the sera and spleens of mice immunized with rSC0016 or rSC0011 carrying either the empty vector pYA3493 or the SaoA-expressing plasmid pS-SaoA than in those with rSC0018 (Figures 5A–C; ^#^ *P* < 0.05, ^##^ *P* < 0.01). rSC0011 with pYA3493 or pS-SaoA induced similar levels of IL-4 to those of rSC0018 with pYA3493 or pS-SaoA (Figure 5B). Higher levels of IFN-γ, IL-4, and IL-17A were observed in the sera and spleens of mice immunized with rSC0016(pS-SaoA) compared to those in mice immunized with rSC0011(pS-SaoA) (Figures 5A–C; ^#^ *P* < 0.05, ^##^ *P* < 0.01). Alternatively, the level of IL-17A in the spleens of mice immunized with rSC0016(pS-SaoA) was similar to rSC0011(pS-SaoA) (Figure 5C). The levels of IFN-γ, IL-4, and IL-17A that were induced by rSC0016(pYA3493) were significantly higher than those induced by rSC0011(pYA3493) in the sera at 3, and 5 days after the booster, as well as in the spleen (Figures 5A–C; ^#^ *P* < 0.05, ^##^ *P* < 0.01). These results indicate that the vector rSC0016 with the Δ*sopB* mutation induced enhanced responses more than the rSC0011 strain did [35].

### *Salmonella* Choleraesuis vaccine vector with *sopB* mutation confers protection against SS2 in mice

When the immunized mice were challenged with 25 times the LD_50_ of wild-type SS2, 100% protection was observed in the mice immunized with strain rSC0016(pS-SaoA), 40% with strain rSC0011(pS-SaoA), and 0% with rSC0018(pS-SaoA) (Table 2). All the mice immunized with BSG or the strain containing the empty vector pYA3493 died at 2–3 days after challenge. These results suggested that the rSC0016(pS-SaoA) strain with the Δ*sopB* mutation conferred significantly greater protection than rSC0011(pS-SaoA) or rSC0018(pS-SaoA) (Table 2; **P < 0.01).

**Table 2:** **Vaccine strains carrying plasmid pS-SaoA confer protection against i.p. challenge with SS2 in BALB/c mice**

Groups	Genotypes	Percentage of survival (Number of mice Survival/Total)^a^
rSC0018(pS-SaoA)	Δ*asdA33*	0% (0/10)
rSC0018(pYA3493)	Δ*asdA33*	0% (0/10)
rSC0016(pS-SaoA)	ΔP_crp527_::TT *araC* P_BAD_ *crp* Δ*pmi*-*2426* Δ*relA199::araC* P_BAD_ *lacI* TT Δ*sopB1686* Δ*asdA33*	100%** (10/10)
rSC0016(pYA3493)	ΔP_crp527_::TT *araC* P_BAD_ *crp* Δ*pmi*-*2426* Δ*relA199::araC* P_BAD_ *lacI* TT Δ*sopB1686* Δ*asdA33*	0% (0/10)
rSC0011(pS-SaoA)	ΔP_crp527_::TT *araC* P_BAD_ *crp* Δ*pmi*-*2426* Δ*relA199::araC* P_BAD_ *lacI* TT Δ*asdA33*	40% (4/10)
rSC0011(pYA3493)	ΔP_crp527_::TT *araC* P_BAD_ *crp* Δ*pmi*-*2426* Δ*relA199::araC* P_BAD_ *lacI* TT Δ*asdA33*	0% (0/10)
BSG	NA	0% (0/10)

** *P* < 0.01 for the survival of mice immunized with rSC0016(pS-SaoA) compared with survival of mice immunized with rSC0011(pS-SaoA) or BSG.

^a^Mice were challenged by i.p. with 25 × LD_50_ of SS2 at 7 weeks after immunization; This experiment was performed twice with same results. The data showed the result of the single experiment.

### *Salmonella* Choleraesuis vaccine vector with *sopB* mutation induces higher antibody responses in piglets

**Figure 6:**
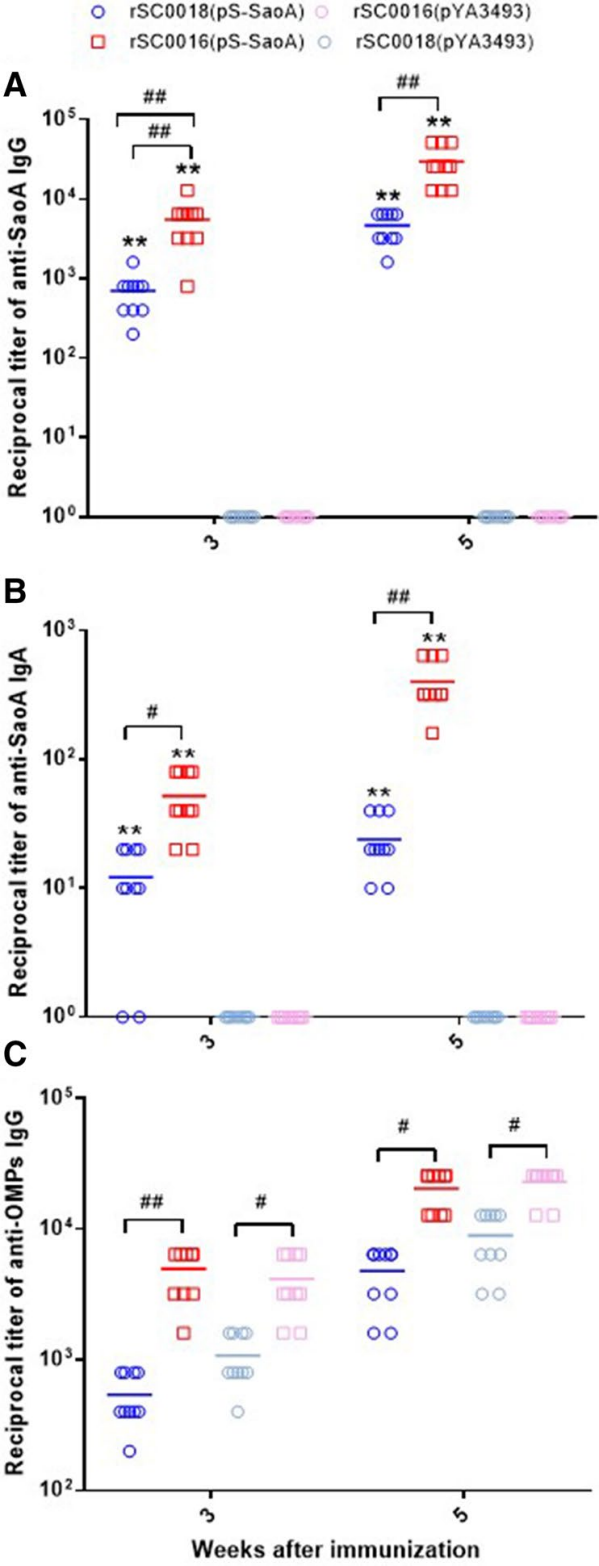
**Serum IgG responses to SaoA** (**A**)**, nasal IgA responses to SaoA** (**B**)**, serum IgG responses to** ***Salmonella*** **Choleraesuis OMPs** (**C**) **in ten pigs were measured by ELISA.** The data represent reciprocal anti-IgG antibody titers from the piglets orally immunized with attenuated *Salmonella* carrying either pS-SaoA and pYA3493 at the indicated weeks after immunization. Serum and nasal cavity wash obtained from individual pig were serially diluted to obtain titers, starting from either 1:50 or 1:10. Error bars represent variation between different pigs. Significant differences were indicated. **P < 0.01, for *Salmonella* Choleraesuis with pS-SaoA compare to *Salmonella* Choleraesuis with pYA3493 in **A** and **B**; ^#^ *P* < 0.05; ^##^ *P* < 0.01, for *Salmonella* carrying either pS-SaoA or pYA3493 compared each other. No responses were detected to antigen tested in pigs immunized with BSG or in pre-immune sera from vaccinated piglets. ELISA was performed twice with identical results.

The immune responses to rSC0016(pS-SaoA) were tested in the piglets. The antibody responses to SS2 SaoA and *Salmonella* OMPs were measured at 3 and 5 weeks after the primary immunization (Figures 6A, B and C). Higher serum IgG and mucosal IgA titers against SaoA were observed at 3 and 5 weeks post-immunization in the swine immunized with rSC0016(pS-SaoA) or rSC0018(pS-SaoA) compared to those with BSG or the empty vector pYA3493 (Figures 6A and B; **P < 0.01). No antibody responses against SaoA were observed in the swine immunized with rSC0016(pYA3493), rSC0018(pYA3493) (Figures 6A and B), or BSG. Notably, the piglets immunized with rSC0016(pS-SaoA) displayed significantly higher serum IgG and mucosal IgA titers against SaoA compared to those with rSC0018(pS-SaoA) (Figures 6A and B; ^#^ *P* < 0.05, ^##^ *P* < 0.01). Similarly, the titers of antibodies directed against *Salmonella* Choleraesuis OMPs induced in pigs by rSC0016(pS-SaoA) or rSC0016(pYA3493) were higher than those by rSC0018(pS-SaoA) or rSC0018(pYA3493), respectively (Figure 6C; ^#^ *P* < 0.05, ^##^ *P* < 0.01), indicating that rSC0016 induces stronger immune responses than did rSC0018.

### *Salmonella* Choleraesuis vaccine vector with *sopB* mutation induces higher levels of IFN-γ, IL-4, and IL-17A in piglets

**Figure 7:**
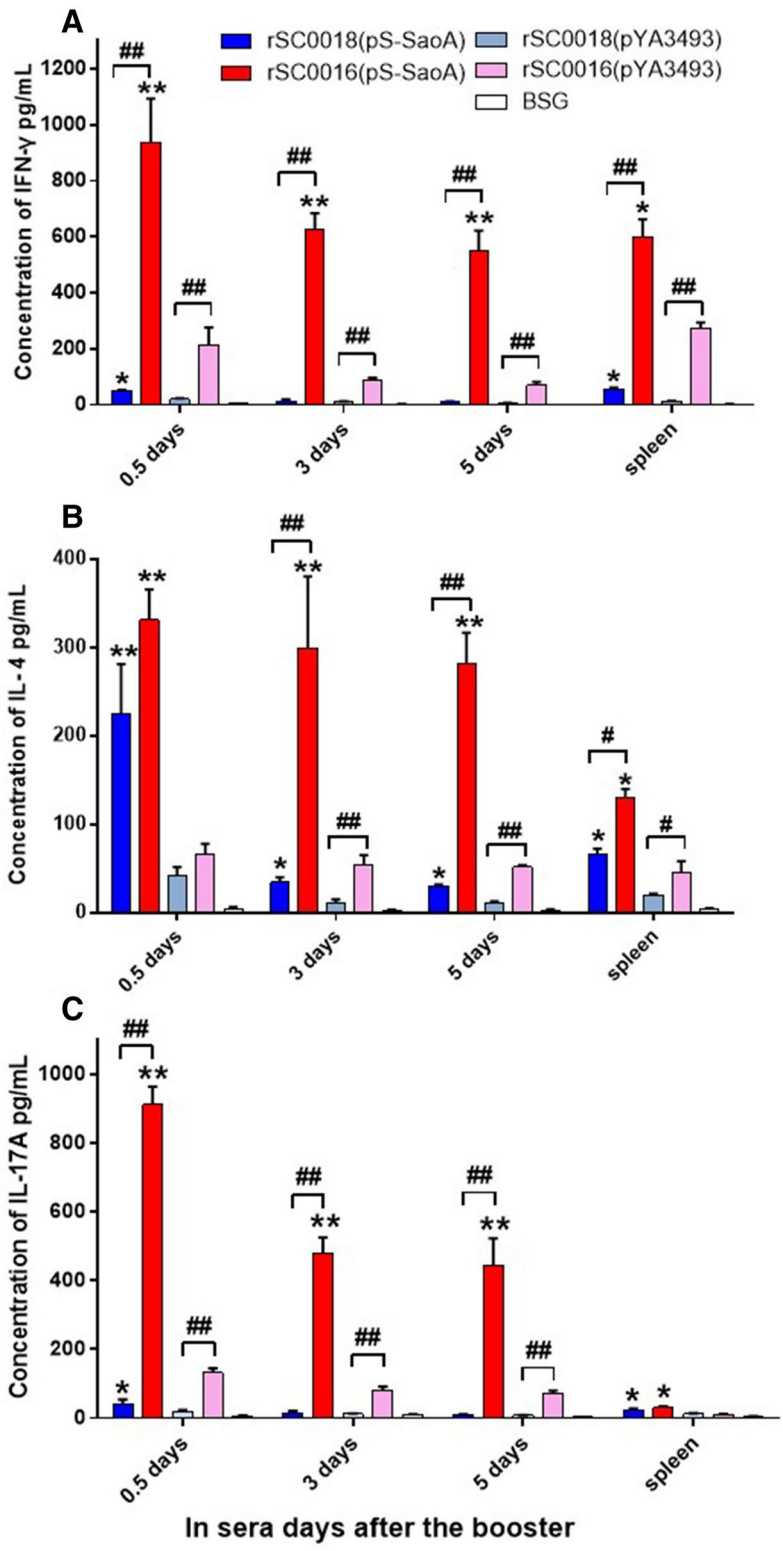
**Cytokine levels in ten pigs. IFN-γ** (**A**)**, IL-4** (**B**) **or IL-17A** (**C**) **in sera from 0.5, 3, and 5** **days, in the spleen at 7** **days after the booster from single piglet were assayed with ELISA.** BSG controls were also included. *P < 0.05; **P < 0.01, for the cytokines levels induced by strains rSC0011, rSC0016 and rSC0018 containing pS-SaoA compared with that containing the empty vector pYA3493; ^#^ *P* < 0.05, ^##^ *P* < 0.01 for significant differences between groups were indicated. The assay was performed in triplicate. The data were collected from two experiments and analyzed.

To further characterize the immune trends in piglet immunized with strain rSC0016 delivering a heterologous antigen, the levels of cytokines IFN-γ, IL-4, and IL-17A in the sera of immunized piglets at 0.5, 3, and 5 days, and in the spleens at 7 days after a boost were tested (Figures 7A–C). Significantly higher levels of IFN-γ, IL-4, and IL-17A were observed in the piglets immunized with the strains rSC0018 and rSC0016 carrying either pS-SaoA or the empty vector pYA3493 compared to those with BSG group (Figures 7A–C; *P* < 0.01). Higher levels of IFN-γ, IL-4, and IL-17A were also detected in the swine immunized with strains rSC0018 and rSC0016 carrying pS-SaoA compared to the strains carrying the empty vector pYA3493 (Figures 7A–C; *P < 0.05, **P < 0.01). Conversely, the levels of IFN-γ and IL-17A in the sera of swine immunized with rSC0018(pS-SaoA) were equal to those immunized with rSC0018(pYA3493) at 3 and 5 days after the booster. Significantly higher levels of IFN-γ, IL-4, and IL-17A were detected in the piglets immunized with rSC0016 carrying either pS-SaoA or pYA3493 compared to those with rSC0018 (Figures 7A–C; ^#^ *P* < 0.05, ^##^ *P* < 0.01), except in the serum at 12 h after the booster. Furthermore, the levels of IFN-γ and IL-17A in the swine immunized with rSC0016(pS-SaoA) were 50-fold higher in the sera 5 days after the booster compared to those with rSC0018(pS-SaoA) (Figures 7A and D). IL-17, mainly produced by Th17 cells [53–55], can synergize with IFN-γ to enhance the production of proinflammatory cytokines [56]. Our results indicated that the new live attenuated strain rSC0016 is more immunogenic than the chemically attenuated strain rSC0018.

### *Salmonella* Choleraesuis vaccine vector with *sopB* mutation confers protection against SS2 in piglets

**Figure 8:**
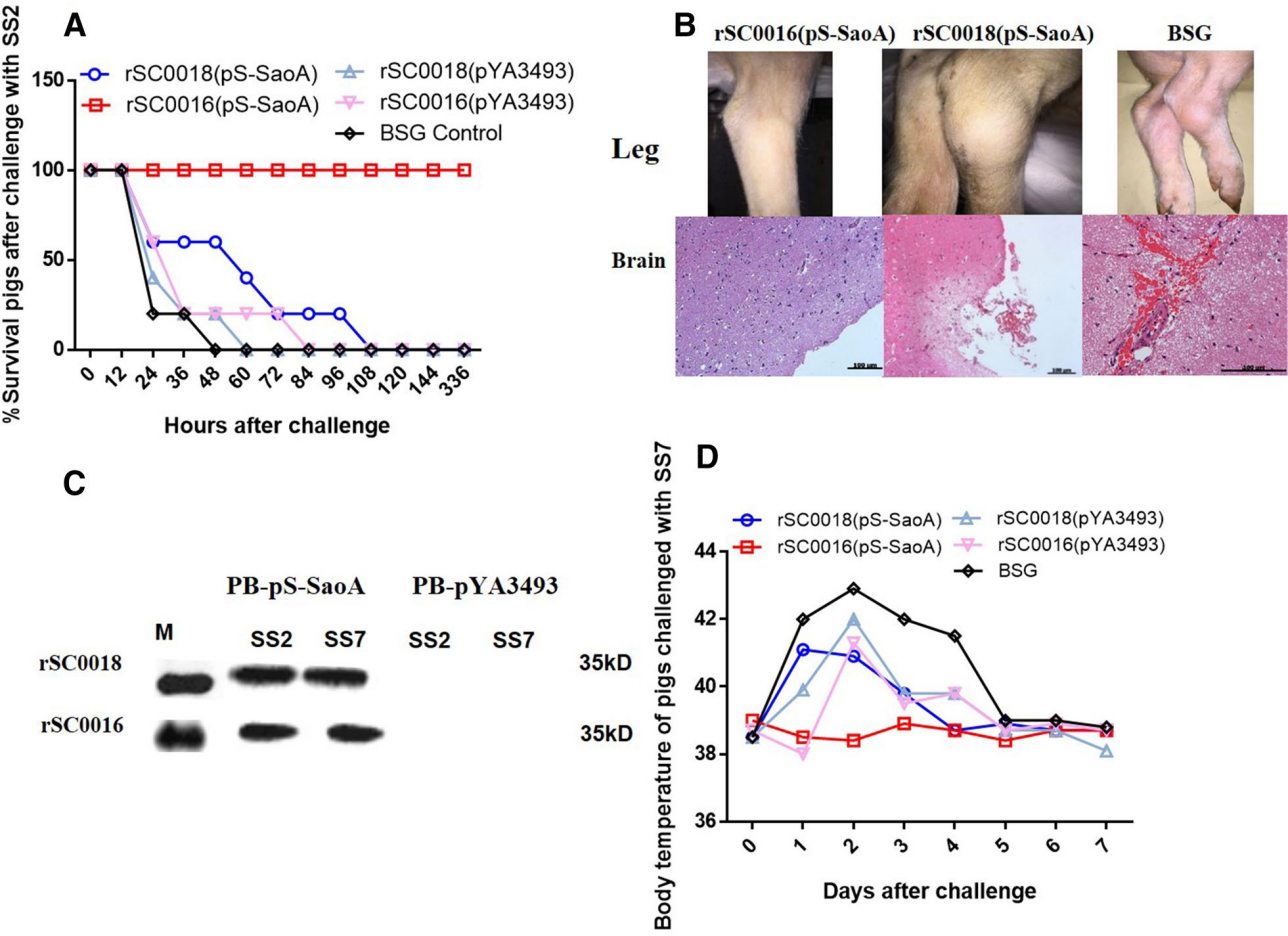
**Protection in pigs. A** Survival curve of swine after challenge with SS2. Groups of ten pigs were orally immunized twice at 3-week intervals with indicated strains and challenged intravenously with 10^8^ CFU of *S. suis* 2 at 2 weeks after the 2^nd^ immunization. (**B**) Clinical symptom of leg and histopathology in the brain of immunized pigs challenged with SS2. (**C**) Western blots analysis the ability of sera from the pigs vaccinated with rSC0016 or rSC0018 containing pS-SaoA plasmid to recognize SaoA protein of SS2 and SS7. PB: Post-boost serum of pigs. (**D**) Rectal temperature of immunized piglets after challenged intravenously with 10^10^ CFU of SS7. The rectal temperatures were measured at 0, 1, 2, 3, 4, 5, 6, and 7 days after challenge. The experiment was performed twice.

Previous studies showed that the percentage of protection conferred by a specific vaccine in pigs is usually lower than that in mice [7, 10, 23]. Therefore, the protective efficacy of the live attenuated strain rSC0016(pS-SaoA) against SS2 was evaluated in piglets. It provided 100% protection against 25 times the LD_50_ of SS2 challenge in mice. Following challenge with 5 × 10^8^ CFU of SS2 (8.9 × LD_50_ in piglets) via the ear vein, all the pigs immunized with either rSC0018(pYA3493) or BSG died within 48 h of challenge (Figure 8A). The piglets immunized with rSC0016(pYA3493) survived longer but died at 84 h after challenge (Figure 8A). The swine immunized with rSC0018(pS-SaoA) died 108 h after challenge and showed symptoms of breathing difficulty and joint swelling (Figures 8A and B). In contrast, all the swine immunized with strain rSC0016(pS-SaoA) survived throughout a 14-day observation period and appeared healthy throughout this period, except for temporary depression within 48 h of a challenge. A histopathological analysis of the challenged swine immunized with either strain rSC0018(pS-SaoA) or BSG showed hemorrhage, congestion, and inflammatory exudation in the brain (Figure 8B). The swine immunized with strain rSC0016(pS-SaoA) showed normal structures (Figure 8B). These results suggest that strain rSC0016(pS-SaoA) provided excellent protection against SS2 in the piglet model.

### *Salmonella* Choleraesuis vaccine vector with *sopB* mutation confers cross-protection against SS7 in piglets

Sao is a highly conserved protein in *S*. *suis* [16]. The protection against SS7, one of the main *S*. *suis* serotypes in China, conferred by rSC0016(pS-SaoA) was evaluated in piglets. SS7 is not lethal in pigs and only led to a fever in pigs after intravenous challenge. The cross-reactivity of the sera from pigs immunized with *Salmonella* Choleraesuis vector rSC0016, carrying SS2 SaoA protein, against SS7 SaoA protein was tested by Western blot. The results showed that the sera from the pigs vaccinated with rSC0016 or rSC0018, carrying SS2 SaoA protein, could recognize the SS7 SaoA protein as well as the SS2 SaoA protein (Figure 8C).

At days 1–4 after challenge with SS7, the swine immunized with rSC0016(pYA3493), rSC0018(pYA3493), rSC0018(pS-SaoA), or BSG showed severe fever (Figure 8D), drowsiness, and asthma. All these symptoms disappeared 6 days after challenge. In contrast, the swine immunized with rSC0016(pS-SaoA) showed no symptoms during the 7-day observation period.

## Discussion

Recently, an innovative *Salmonella* Choleraesuis live attenuated vaccine vector rSC0011 with the mutations for regulated delayed attenuation (Δ*pmi*-*2426* deletion mutation, ΔP_crp527_::TT *araC* P_BAD_ *crp* deletion-insertion mutation) [29, 33], and regulated delayed antigen synthesis (Δ*relA*::*araC* P_BAD_ *lacI* TT deletion-insertion mutation) was developed [29, 57]. This vaccine vector, delivering a conserved protein of *S. suis*, only conferred protection against a low-dose challenge of SS2 in mice [34], but not a high-dose challenge of SS2 (Table 2). Therefore, it was necessary to improve the immunogenicity of the strain, rSC0011. The SopB protein of *Salmonella* Typhimurium plays an immunosuppressive role [48], and its inactivation improves humoral and cellular immune responses of the host [35, 48]. In this study, a *sopB* deletion mutation was introduced into our *Salmonella* Choleraesuis vector with the regulated delayed attenuation system to generate the new strain rSC0016. This new strain displayed the expected phenotypes of reduced fluid secretion and inflammation in rabbit ileal loop test, indicating that the Δ*sopB* deletion in *Salmonella* Choleraesuis had the same effect as it had in other *Salmonella* serotypes [52, 58]. Strain rSC0016 showed a similar distribution to that of strain rSC0011 in Peyer’s patches of mice, confirming that the *sopB* mutation did not affect the gut colonization of the strain [48, 52]. However, the bacterial load of rSC0016 in the spleen was significantly lower than that of rSC0011. This result indicates that strain rSC0016 is more attenuated than rSC0011 in deep lymphoid tissue.

Our results demonstrated that strain rSC0016, with the Δ*sopB* mutation, induces higher levels of IgA, IgG, IFN-γ, IL-4 γ and IL-17A in mice than the other strains tested. These results may relate to the immunosuppressive function of *sopB*. In addition, it might also be related to the higher antigen loads of rSC0016 in the tissues than those of rSC0018. When *Salmonella* enters the cell, 10–20% of the bacteria remain in the cytosol, whereas the majority of the internalized bacteria remain within a modified phagosome, the *Salmonella*-containing vacuole [59, 60]. The cytosolic bacteria replicate faster than the vacuolar bacteria and are invasion-primed and -competent [60]. A small but significant amount of the cytosolic bacteria can leave epithelial cells, facilitating a rapid secondary infection and inducing both enteric and systemic infections [60]. The *sopB* deletion mutant does not affect the intracellular replication of the bacterium but does induce the early death of these cells, increases lysis, and promotes the transmission of the bacteria [59]. The premature death of epithelial cells exposes the strain to the immune system at a great efficiency, which may contribute to the increased immune responses triggered by rSC0016 compared to rSC0011.

The *Salmonella* Choleraesuis C500 vaccine induces mucosal IgA and IFN-γ responses during its protection of pigs [61], whereas our strain rSC0016 induced greater IgA, IgG, IFN-γ, IL4, and IL17A responses compared to strain rSC0018, which was derived from C500. IL-17A induces and mediates proinflammatory responses and the expression of many other cytokine genes. The higher levels of IL-17A detected in the sera (34–50 fold) of swine immunized with rSC0016 compared to those with rSC0018 may help the swine clear the infecting pathogen and confer greater protective immunity. The detailed mechanism underlying high IL-17A levels induced by rSC0016 in pig warrants further study.

The immune responses induced by strains rSC0016 and rSC0018 in both mice and weaned piglets were compared. In mice, the antibody titers of IgG and IgA against Sao of *S*. *suis* and IgG against the OMPs of *Salmonella* Choleraesuis induced by rSC0016 were significantly higher than those induced by rSC0011. Both were higher than those induced by rSC0018, indicating that the immune responses induced by the combination of the regulated delayed strategy and the *sopB* mutation were superior to those induced by the regulated delayed strategy alone or with chemically attenuated rSC0018. The trends in the immune responses of weaned piglets were similar to those of mice. It is noteworthy that the mucosal IgA responses induced by rSC0016 were significantly higher than those induced by rSC0011 in mice, and were significantly higher than those induced by rSC0018 in mice or in piglets after immunization. Our orally delivered *Salmonella* vaccine vectors induced strong mucosal and humoral responses against the Sao antigen (Figures 4 and 6). IgA could prevent the adhesion of pathogens to mucosal cells and improve the protection efficiency of vaccine strains. However, current challenge models (i.p. in mice and i.v. in pig) did not address this. Current challenge models focused on the effectiveness of our candidate vaccine against septicemia, since both i.p. and i.v. challenge could induce severe sepsis or even death. If a vaccine candidate could protect the animal from more severe septicemia, it will reduce more severe consequence, even the host is infected with *S. suis*. Since Sao is not a major virulence factor [62], IgG response is important for the opsonophagocytic killing of *S. suis* [24]. The role of IgA in the protection against *S. suis* needs to be studied.

Globally, the predominant *S*. *suis* serotypes isolated from clinical cases of the disease in pigs are serotypes 2, 9, 3, 1/2, and 7 [63]. Preventing the diseases caused by *S*. *suis* in swine with a universal vaccine is a long-sought goal. The Sao protein is highly conserved in *S. suis* [16]. However, cross-protection against serotypes 1 and 7, in addition to SS2, have only been reported in mice [19, 64]. Whether the Sao protein delivered by *Salmonella* Choleraesuis could be a universal vaccine for swine against other *S*. *suis* serotypes is not known. Our results showed that the sera from the pigs vaccinated with rSC0016 or rSC0018 carrying SS2 SaoA protein recognized the SS7 SaoA protein, which shared 95.2% amino acid homology with SS2 SaoA (data not show). Piglets immunized with rSC0016(pS-SaoA) had normal body temperatures after challenge with SS7, whereas pigs immunized with either rSC0018(pS-SaoA), a control strain carrying the empty vector, or BSG experienced fever 1–4 days after challenge. This indicates that SaoA delivered by the innovative *Salmonella* Choleraesuis vector, which combines the regulated delayed strategy and the *sopB* mutation, provides cross-protection against SS7.

In conclusion, the new recombinant attenuated *Salmonella* Choleraesuis vector, which combines the regulated delayed strategy and the *sopB* mutation, displays a balance between adequate attenuation and safety, and induces improved immune responses. The new recombinant attenuated *Salmonella* Choleraesuis strain, synthesizing the SaoA protein, provided full protection against SS2 in mice and pigs. SS7 is not lethal and only leads to fever in pigs by intravenous injection without displaying other clinical signs. Vaccinated pigs did not develop fever after challenge with SS7, indicating that our vaccine is cross-protective against SS7 in swine. In the future, the protection afforded by the new recombinant attenuated *Salmonella* Choleraesuis vector against other serotypes of *S. suis*, such as serotypes 9, 3, and 1/2 will be evaluated in pigs. The data reported in this study laid the foundation for the development of a universal vaccine against *S. suis*.
